# EHMTI-0270. Experience with onabotulinumtoxin type a in high-frequency episodic migraine

**DOI:** 10.1186/1129-2377-15-S1-G30

**Published:** 2014-09-18

**Authors:** P Pozo-Rosich, M Quintana, J Alvarez-Sabin, N Mas Sala

**Affiliations:** 1 Neurology, Hospital Universitari Vall d'Hebron, Barcelona, Spain

## 

To analyse the efficacy of onabotulinumtoxin type A (OnabotA) in High Frequency Episodic Migraine (HFEM) after two treatment cycles.

Twenty-three consecutive patients (22 women, 1 man) diagnosed with HFEM and with an inadequate response or intolerance to oral preventatives were included. Data regarding frequency and intensity of headache attacks, analgesic use and migraine disability assessment scale (MIDAS) before and after OnabotA was collected. The current oral preventive therapy was continued in all patients.

Good responders were those who after treatment improved from HFEM to low-frequency episodic migraine, presented with a reduction in 75% of headpain intensity, had a reduction of the oral analgesic/triptans use to one or less per week, and improved from a severe to a mild disability measured using the MIDAS scale (improvement in four categories). This was achieved in 16 patients (69.6%). Six other patients (26%) presented with a partial reponse to OnabotA, this was measured by an improvement from severe to moderate or minimal disability measured using the MIDAS scale and a reduction in the analgesic use (improvement in two categories). One patient did not improve after treatment.

In our clinical practice, treatment of refractory HFEM with OnabotA reduces the frequency and the intensity of the migraine attacks, reduces the consumption of symptomatic treatments and, improves the disability related with migraine. This makes it a good preventive therapeutic option.

